# Prognostic impact of CD57, CD68, M-CSF, CSF-1R, Ki67 and TGF-beta in soft tissue sarcomas

**DOI:** 10.1186/1472-6890-12-7

**Published:** 2012-05-03

**Authors:** Sveinung W Sorbye, Thomas K Kilvaer, Andrej Valkov, Tom Donnem, Eivind Smeland, Khalid Al-Shibli, Roy M Bremnes, Lill-Tove Busund

**Affiliations:** 1 Dept of Clinical Pathology, University Hospital of North Norway, Tromso, 9038, Norway; 2 Institute of Medical Biology, University of Tromso, Tromso, Norway; 3 Dept of Oncology, University Hospital of North Norway, Tromso, Norway; 4 Institute of Clinical Medicine, University of Tromso, Tromso, Norway; 5 Dept of Pathology, Nordland Central Hospital, Bodo, Norway

**Keywords:** Soft tissue sarcomas, STS, Malignancy grade, DSS, Macrophages, NK cells, CD57, Ki67, TGF-beta, TMA

## Abstract

**Background:**

Prognostic markers in curable STS may have the potential to guide therapy after surgical resection. The purpose of this study was to clarify the prognostic impact of the presence of cells and growth factors belonging to the innate immune system in soft tissue sarcomas (STS). The significance of macrophages (CD68), their growth factor macrophage colony-stimulating factor (M-CSF), its receptor colony-stimulating factor-1 receptor (CSF-1R), natural killer cells (CD57) and the general immunomodulating molecule (TGF-beta) are all controversial in STS. Herein, these markers are evaluated and compared to the cell proliferation marker Ki67.

**Methods:**

Tissue microarrays from 249 patients with non-gastrointestinal (non-GIST) STS were constructed from duplicate cores of viable and representative neoplastic tumor areas and duplicate cores of peritumoral capsule. Immunohistochemistry was used to evaluate the expression of CD68, M-CSF, CSF-1R, CD57, TGF-beta and Ki67 in tumor and peritumoral capsule.

**Results:**

In univariate analyses increased expression of M-CSF (P = 0.034), Ki67 (P < 0.001) and TGF-beta (P = 0.003) in tumor correlated with shorter disease-specific survival (DSS). Increased expression of CD68 in tumor correlated significantly with malignancy grade (P = 0.016), but not DSS (P = 0.270). Increased expression of Ki67 in peritumoral capsule tended to correlate with a shorter DSS (P = 0.057). In multivariate analyses, co-expression of M-CSF and TGF-beta (P = 0.022) in tumor and high expression of Ki67 (P = 0.019) in peritumoral capsule were independent negative prognostic factors for DSS.

**Conclusions:**

Increased co-expression of M-CSF and TGF-beta in tumor in patients with STS, and increased expression of Ki67 in peritumoral capsule were independent negative prognostic factors for DSS.

## Background

Soft tissue sarcomas (STS) are heterogeneous malignancies originating from the mesenchymal lineage. There are more than 50 different histological entities and they comprise less than 1% of adult malignancies [1]. The STS are among the most aggressive cancer types with a lethality of 40–50% due to metastasis or local relapse [2]. There are several prognostic factors which determine tumor progression, and ultimately the patient’s outcome, including positive resection margins, presence of local recurrence, histological entity and tumor grade, size, location and depth [3-9].

Many studies have been designed to investigate the prognostic factors of STS by using immunohistochemical methods [10], and most of the published data have focused on the expression of markers for cell kinetics and regulatory proteins of the cell cycle.

Immunotherapy and vaccines with the capability to activate the host immune system may have a role as second-line therapy, and characterization of the in situ cellular and molecular immunology form the basis for such therapy [11]. Hence, clinical data on the prognostic significance of different immunological cells are warranted.

The innate immune system consists mainly of granulocytes, macrophages, natural killer (NK) cells, dendritic cells (DCs) and their corresponding growth factors and receptors [12]. They mediate major histocompatibility complex unrestricted cytotoxicity and are essential in the immediate limitation and elimination of foreign challenges to the host, including defense against cancer, but lack the ability of ‘memory’ when re-exposed to the same antigen[12,13]. The NK cell has a well-established role in tumor rejection in a variety of cancers[14-16], and the mechanism by which these cells discriminate tumor from normal cells has provided new insights into tumor immunosurveillance and has suggested new strategies in the treatment of human cancer [17,18].

Ki67 expression increases with increasing malignancy grade in many cancer types of different lineages [19-23]. In Ewing’s sarcoma, high Ki67 expression was an independent prognostic factor for progression free survival and overall survival independent of treatment type [24].

We have previously reported the prognostic significance of the humoral immune system by lymphocyte infiltration in tumor [25] and peritumoral capsule [26] of STS. We have also reported the significance of the innate immune system by the correlation of expression of macrophages (CD68), their growth factor macrophage colony-stimulating factor (M-CSF), its receptor colony-stimulating factor-1 receptor (CSF-1R) and histological grade in STS [27]. It was important to validate these findings in a different material, explore the relationship to expression of Ki67, disease-specific survival (DSS) and include other markers as CD57 and TGF-beta. The purpose of this study was to examine the prognostic role of the innate immune system in STS by assessing the expression of CD68, M-CSF, CSF-1R, CD57, TGF-beta and Ki67.

## Methods

### Patients and clinical samples

The National Cancer Data Inspection Board and The Regional Committee for Research Ethics approved the study. The material was collected from our approved biobank for paraffin embedded material and slides. Data were analyzed anonymously.

Primary tumor tissue from untreated patients diagnosed with STS at the University Hospital of North Norway (UNN) from 1973 to 2006 and the Hospitals of Arkhangelsk region, Russia, from 1996 to 2006 was used in this retrospective study. 496 potentially suitable patient records were identified from the hospital database, but only 249 of these were eligible based on complete medical records and adequate paraffin-embedded tissue blocks. In 80 of these cases it was also possible to obtain tissue from the peritumoral capsule for TMA [26]. This report includes follow-up data for 167 Norwegian and 82 Russian patients until September 2009. The median follow-up was 38 (range 0–392) months.

The histology of all soft tissue sarcoma cases was reviewed according to modern classification (WHO, 2002) by two dedicated pathologists (AV and SWS). For the Russian material, new slides were made from all paraffin blocks. For the Norwegian material, new slides were made when necessary. All biopsies were immunostained with cytokeratin (CK), c-kit (CD117), Actin, smooth muscle actin (SMA), vimentin (VIM) and CD34. Some slides were also stained with S100 if necessary to rule out differential diagnoses. Further molecular methods were, in general, not considered necessary for differential diagnostics, but in some cases PCR or FISH were performed. About 10% of the initial diagnoses were revised due to altered classification and the appearance of new entities such as GIST. All carcinosarcomas, endometrial sarcomas, carcinomas and lymphomas were excluded.

### Microarray construction

Tissue microarrays (TMAs) were constructed for high-throughput molecular pathology research[28-30]. The slides were evaluated by two pathologists (AV and SWS) using light microscope to identify the tumor and the peritumoral capsule. The most representative areas of the tumor and peritumoral capsule were carefully selected and marked on the hematoxylin and eosin (HE) slides for the corresponding donor blocks and sampled for the tissue microarray collector blocks[26]. The TMAs were assembled using a tissue-arraying instrument (Beecher Instruments).

Studies suggest that punching multiple 0.6 mm cores from different regions captures the heterogeneity of the capsule more accurately than single 2 to 4 mm cores [30]. Hence, we obtained two 0.6-mm cores of tumor and two cores from peritumoral capsule (four cores from each patient). These were secured from different representative areas of the tissue block and selected to be as representative as possible. To include all core samples, 12 tissue array blocks were constructed. Multiple 4-μm sections were cut with a Micron microtome (HM355S) and specific antibodies were stained for immunohistochemistry (IHC).

### Immunohistochemistry (IHC)

Sections were deparaffinized with xylene and rehydrated with ethanol. Antigen retrieval was performed by placing the specimens in 0.01 M citrate buffer at pH 6.0 and exposing them to two repeated microwave heatings of 10 min at 450 W. The slides were then transferred to the Ventana Benchmark, XT automated slide stainer (Ventana Medical System, Illkirch, France). Tissue sections were incubated with primary mouse monoclonal antibodies recognizing Ki67, CD68 and CD57 (Ventana Medical System), as well as rabbit polyclonal M-CSF, CSF-1R (clone H-300; Santa Cruz Biotechnology, Santa Cruz, CA, USA) and TGF-beta (clone sc-146; Santa Cruz). The dilution was 1:5 for M-CSF, 1:25 for CSF-1R and 1:50 for TGF-beta. All Ventana antibodies were prediluted by the manufacturer. The incubation periods were 16 min for Ki67, CD57 and CD68, and 28 min for TGF-beta, M-CSF and CSF-1R. As secondary antibodies, biotinylated goat antimouse IgG and mouse antirabbit IgM, both 200 lg ml, were used. The Dako EnVision + System-Horseradish Peroxidase [diaminobenzidine (DAB)] kit (Dako, Glostrup, Denmark) was used to block endogenous peroxidase. This was followed by application of liquid diaminobenzidine as substrate-chromogen, yielding a brown reaction product at the site of the target antigen (iView DAB® procedure). Finally, slides were counterstained with hematoxylin to visualize the nuclei. For each antibody, including controls, all TMA staining were performed in a single experiment. As negative staining controls, the primary antibodies were replaced with the primary antibody diluents. In the TMA we also used cores from carcinomas and normal tissue as positive and negative controls.

### Scoring of IHC

**Figure 1:**
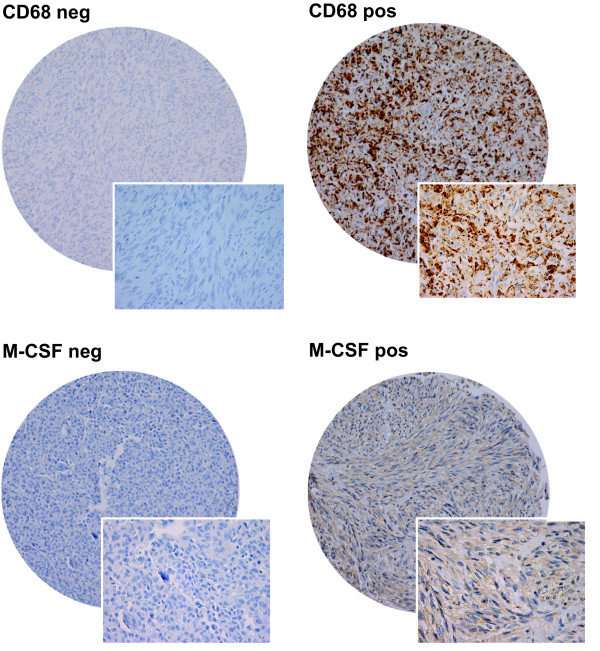
**IHC microscopic pictures of TMA of soft tissue sarcoma representing different expression of CD68 and M-CSF.** (A) CD68 low expression; (B) CD68 high expression; (C) M-CSF low expression; (D) M-CSF high expression. Original magnification X 100 and 400.

The ARIOL imaging system (Genetix, San Jose, CA) was used to scan the slides for antibody staining of the TMAs[26]. The number of CD57 positive cells (including NK cells) and CD68 positive cells (including macrophages) in tumors were scored as 0 (no cells), 1 (1–5 cells), 2 (6–19) or 3 (20+ cells) per 0.6 mm core. Examples are shown in Figure 1. Regarding M-CSF, CSF-1R, Ki67 and TGF-beta, expression was scored as: 0, negative; 1, weak; 2, intermediate; and 3, strong. The mean score from the duplicate cores from tumor or capsule, respectively, was used. Marker expression was dichotomised (low vs. high), and high expression defined as mean score ≥ 0.30 for CD68, ≥ 0.75 for TGF-beta, ≥ 2.00 for Ki67 and ≥ 0.01 for CD57, M-CSF and CSF-1R. All samples were anonymized and independently scored by two pathologists (AV and SWS). When disagreement, the slides were re-examined and consensus was reached by the observers. When assessing a variable for a given score, the scores of the other variables and the outcome were hidden from the observers.

### Statistical methods

All statistical analyses were done using the statistical package SPSS (Chicago, IL), version 18. The immunohistochemistry scores from each observer were compared for interobserver reliability by use of a two-way random effect model with absolute agreement definition. The intraclass correlation coefficient (reliability coefficient) was obtained from these results.

The Chi-square test and Fishers Exact test were used to examine the association between molecular marker expression and various clinicopathological variables. Univariate analyses were performed using the Kaplan-Meier method, and statistical significance between survival curves was assessed by the log rank test. Disease-specific survival (DSS) was determined from the date of confirmed STS diagnosis.

The multivariate analysis was carried out using the Cox proportional hazards model to assess the independent impact of each pre-treatment variable on survival in the presence of other variables. Only significant variables from the univariate analyses were entered into the Cox regression analysis. Probability for stepwise entry and removal was set at 0.05 and 0.10, respectively. The significance level used was p < 0.05.

## Results

### Clinicopathological variables

Demographic, clinical, and histopathological variables are shown in Table 1. Patient age range was 0–91 years (mean 55 years), and 44% of the patients were males. The non-GIST STS comprised 68 undifferentiated pleomorphic sarcoma, 67 leiomyosarcoma, 34 liposarcoma, 20 malignant fibroblastic/myofibroblastic tumors, 16 rhabdomyosarcoma, 16 synovial sarcoma, 13 angiosarcoma, 11 malignant peripheral nerve sheath tumors (MPNST) and 4 other STS. There were 61 low grade STS (24%) and 188 high grade (FNCLCC grade 2 and 3) STS (76%).

**Table 1:** Prognostic clinicopathological variables as predictors for disease-specific survival soft tissue sarcomas (univariate analysis, log rank test), N = 249

**Characteristic**		**Patients (n)**	**Patients (%)**	**Median survival (months)**	**5-Year survival (%)**	**P**
**Age**						
≤ 20 years		20	8	15	40	0.126
21-60 years		113	45	68	52	
> 60 years		116	47	30	40	
**Gender**						
Male		110	44	41	46	0.390
Female		139	56	45	45	
**Nationality**						
Norwegian		167	67	63	51	0.011
Russian		82	33	22	34	
**Histology**						
Undifferentiated pleomorphic sarcoma		68	27	29	40	0.102
Leiomyosarcoma		67	27	45	46	
Liposarcoma		34	14	NR	67	
MF/MFT		20	8	43	50	
Angiosarcoma		13	5	10	31	
Rhabdomyosarcoma		16	6	17	38	
MPNST		11	4	49	45	
Synovial sarcoma		16	6	31	29	
Other STS		4	2	NR	75	
**Tumor localization**						
Extremities		89	36	100	53	0.348
Trunk		47	29	32	44	
Retroperitoneum		37	25	25	38	
Head/Neck		18	7	15	41	
Visceral		58	23	30	42	
**Tumor size**						
< 5 cm		74	30	127	57	0.027
5-10 cm		91	37	44	45	
> 10 cm		81	32	28	37	
Missing		3	1			
**Malignancy grade FNCLCC**						
1		61	25	NR	74	<0.001
2		98	39	41	45	
3		90	36	16	26	
**Tumor depth**						
Superficial		17	7	NR	93	<0.001
Deep		232	93	36	42	
**Metastasis at time of diagnosis**						
No	206		83	76	53	<0.001
Yes	43		17	10	10	
**Surgery**						
Yes	228		92	59	50	<0.001
No	21		8	5	0	
**Surgical margins**						
Wide	108		43	NR	62	<0.001
Non-wide	141		57	19	33	
**Chemotherapy**						
No	191		77	52	47	0.424
Yes	58		23	29	40	
**Radiotherapy**						
No	176		71	48	46	0.590
Yes	73		29	38	43	

Abbreviations: MF/MFT, malignant fibroblastic/myofibroblastic tumors; MPNST, malignant peripheral nerve sheath tumor; STS, soft tissue sarcomas; NR, not reached; NOS, non specified.

The treatment option of choice was surgery (n = 228), seven patients received chemotherapy and/or radiotherapy only, and 14 patients received no therapy. A total of 120 patients received surgery only, 55 surgery and radiotherapy; 40 surgery and chemotherapy and 13 surgery, radiotherapy and chemotherapy. Two patients received chemotherapy only, three both chemotherapy and radiotherapy, and two radiotherapy only. The 5-year survival with non-wide resection margins was 33% and with wide resection margins 62%.

### Inter-observer variability

There was good reproducibility between the two investigating pathologists. The scoring agreement was tested for M-CSF and CD68 expression in tumor. The IHC scores from each observer were compared using a two-way random effect model with absolute agreement definition. The intraclass correlation coefficients (reliability coefficients, r) obtained from these results were 0.85 for M-CSF (P < 0.001) and 0.90 for CD68 (P < 0.001).

### Univariate analyses

Nationality, tumor size, malignancy grade, tumor depth, metastasis at time of diagnosis, surgery and surgical margins were all significant indicators for disease-specific survival (DSS) in univariate analyses (Table 1).

**Figure 2:**
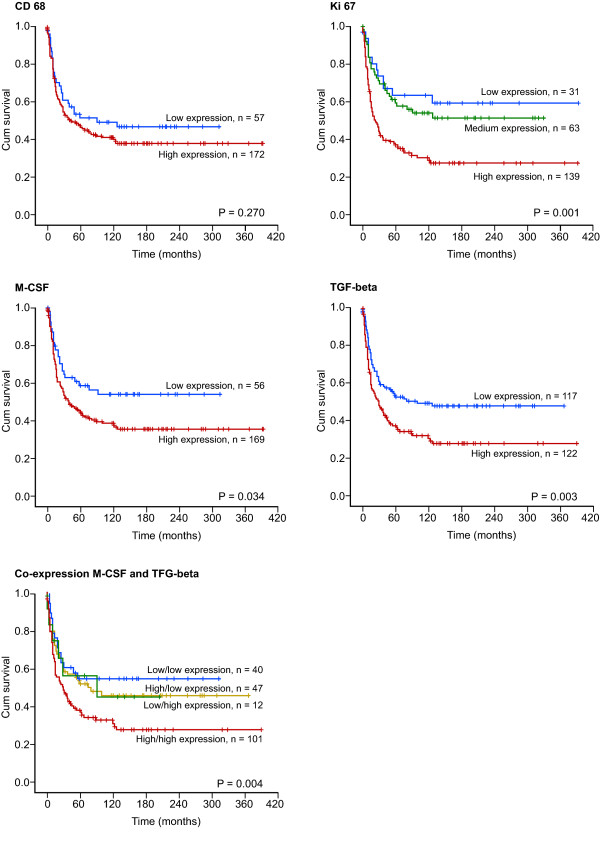
Disease-specific survival curves for high and low expression of CD68, Ki67, M-CSF, TGF-beta and co-expression M-CSF and TGF-beta in tumor in patients with STS (N = 249).

Besides, increased expression of M-CSF (P = 0.034), Ki67 (P < 0.001) and TGF-beta (p = 0.003) in tumor correlated significantly with a shorter DSS, (Table 2 and Figure 2). Co-expression of M-CSF and TGF-beta (p = 0.004) also correlated with shorter DSS. No such relationship was observed for CD57, CD68, and CSR-1R.

**Table 2:** Expression of markers in tumor and their prediction for disease-specific survival in patients with soft tissue sarcomas (univariate analysis; log-rank test), N = 249

**Marker expression**	**Patients (n)**	**Patients (%)**	**Median survival (months)**	**5-Year survival (%)**	**P**
**CD 57**					
Low	93	37	54	49	0.617
High	135	54	49	48	
Missing	21	8			
**CD 68**					
Low	57	23	91	52	0.270
High	172	69	45	47	
Missing	20	8			
**M-CSF**					
Low	56	22	NR	59	0.034
High	169	68	38	44	
Missing	24	10			
**CSF-1R**					
Low	38	15	41	44	0.832
High	191	77	38	46	
Missing	20	8			
**Ki67**					
Low	31	12	NR	63	<0.001
Medium	63	25	NR	59	
High	139	56	24	37	
Missing	16	6			
**TGF-beta**					
Low	117	47	99	53	0.003
High	122	49	29	37	
Missing	10	4			
**M-CSF and TGF-beta**					
Low	119	48	91	53	0.004
High	101	41	29	38	
Missing	29	12			

Abbreviations: NR, not reached.

A shorter DSS with increased expression of M-CSF was seen in females (P = 0.025), Norwegian patients (P = 0.015) and in patients with tumors larger than 5 cm (P = 0.018, data not shown).

Increased expression of Ki67 in the peritumoral capsule correlated with a shorter DSS (N = 80, P < 0.001). Increased expression of CD68 in the peritumoral capsule tended to correlate with a shorter DSS, though not statistically significant (N = 80, P = 0.057), Table 3. No prognostic impact was observed for CD57, M-CSF, CSR-1R, TGF-beta or co-expression of M-CSF and TGF-beta. There was a correlation of expression of Ki67 in tumor (N = 249, P = 0.001) and metastasis at the time of the diagnosis, but not no correlation of expression of Ki67 in peritumoral capsule (N = 80, P = 0.395) and metastasis at the time of the diagnosis (data not shown).

**Table 3:** Expression of markers in peritumoral capsule and their prediction for disease-specific survival in patients with soft tissue sarcomas (univariate analysis; log-rank test), N = 80

**Marker expression**	**Patients (n)**	**Patients (%)**	**Median survival (months)**	**5-Year survival (%)**	**P**
**CD 57**					
Low	50	63	38	47	0.797
High	29	36	123	55	
Missing	1	1			
**CD 68**					
Low	34	43	NR	61	0.057
High	45	56	31	43	
Missing	1	1			
**M-CSF**					
Low	36	45	75	54	0.608
High	39	49	36	46	
Missing	5	6			
**CSF-1R**					
Low	36	45	52	49	0.587
High	37	46	57	47	
Missing	7	9			
**Ki67**					
Low	32	40	NR	74	<0.001
High	37	46	29	35	
Missing	11	14			
**TGF-beta**					
Low	43	54	52	50	0.906
High	28	35	31	50	
Missing	9	11			
**M-CSF and TGF-beta**					
Low	24	30	80	57	0.626
High	42	53	31	45	
Missing	14	18			

Abbreviations: NR, not reached**.**

In co-variation analyses between malignancy grade and expression of the different markers in tumor, Ki67, CD68, M-CSF and TGF-beta showed statistical significance (data not shown). Increased expression of CD68 in tumor correlated with malignancy grade (P = 0.016) and expression of Ki67 (P < 0.001). Increased expression of M-CSF in tumor correlated with malignancy grade (P = 0.010) and expression of Ki67 (P = 0.002). Increased expression of TGF-beta in tumor correlated with malignancy grade (P = 0.029) and expression of Ki67 (P = 0.005), table 4 and 5. There was a co-variation between expression of M-CSF and TGF-beta in tumor (P < 0.001, data not shown). In crosstabulation the expected count in the low M-CSF, high TGF-beta group was 26.7 patients (data not shown), but the observed count was 12 patients (Figure 2).

**Table 4:** Results of expression of CD68 and M-CSF in tumor versus malignancy grade in patients with soft tissue sarcomas, N = 249

**Expression**	**Malignancy grade (%)**			
	Grade 1	Grade 2	Grade 3	Total
CD68, Low	23 (40)	16 (28)	18 (32)	57 (100)
CD68, High	37 (22)	73 (42)	62 (36)	172 (100)
Total	60 (26)	89 (39)	80 (35)	229 (100)
Missing	20		Chi-Square	8.319
			P-value	0.016
	Grade 1	Grade 2	Grade 3	Total
M-CSF, Low	23 (41)	18 (32)	15 (27)	56 (100)
M-CSF, High	35 (21)	67 (40)	67 (40)	169 (100)
Total	58 (26)	85 (38)	82 (37)	225 (100)
Missing	24		Chi-Square	9.300
			P-value	0.010
	Grade 1	Grade 2	Grade 3	Total
TGF-beta, Low	15 (13)	66 (56)	36 (31)	117 (100)
TGF-beta, High	6 (5)	71 (58)	45 (37)	122 (100)
Total	21 (9)	137 (57)	81 (34)	239 (100)
Missing	10		Chi-Square	7.091
			P-value	0.029

**Table 5:** Results of expression of CD68 and M-CSF in tumor versus expression of Ki67 in patients with soft tissue sarcomas, N = 249

**Expression**	**Ki67 (%)**			
	Low	Medium	High	Total
CD68, Low	15 (27)	19 (35)	21 (38)	55 (100)
CD68, High	15 (9)	42 (25)	110 (66)	167 (100)
Total	30 (14)	61 (28)	131 (59)	222 (100)
Missing	27		Chi-Square	16.947
			P-value	<0.001
	Low	Medium	High	Total
M-CSF, Low	14 (26)	16 (30)	23 (43)	53 (100)
M-CSF, High	15 (9)	41 (25)	108 (66)	164 (100)
Total	29 (13)	57 (26)	131 (60)	217 (100)
Missing	32		Chi-Square	12.695
			P-value	0.002
	Low	Medium	High	Total
TGF-beta, Low	21 (18)	34 (30)	59 (52)	114 (100)
TGF-beta, High	7 (6)	28 (25)	79 (69)	114 (100)
Total	28 (12)	62 (27)	138 (31)	228 (100)
Missing	21		Chi-Square	10.749
			P-value	0.005

### Multivariate analyses

Significant demographic, clinicopathological, and expression variables from the univariate analyses were entered into the multivariate Cox regression analysis. In the multivariate analysis, the co-expression of M-CSF and TGF-beta in the tumor was an independent prognostic factor for DSS. Other independent negative prognostic variables were malignancy grade (P < 0.001), metastasis at time of diagnosis (P < 0.001) and non-wide resection margins (P = 0.001, Table 6).

**Table 6:** Results of Cox regression analysis summarizing significant independent prognostic factors in patients with soft tissue sarcomas

	**Tumor, N=249**			**Capsule, N=80**		
**Factor**	**Hazard Ratio**	**95% CI**	**P**	**Hazard Ratio**	**95% CI**	**P**
**Nationality**						
Norwegian	1.000			1.000		
Russian	0.948	0.603-1.490	0.816	0.588	0.263-1.312	0.194
**Tumor size**						
< 5 cm	1.000		0.540*	1.000		0.342*
5-10 cm	1.103	0.687-1.770	0.685	0.888	0.376-2.099	
> 10 cm	1.310	0.797-2.153	0.287	1.671	0.660-4.233	
**Malignancy grade FNCLCC**						
1	1.000		0.001*	1.000		0.051*
2	1.997	1.129-3.531	0.017	1.402	0.383-5.137	0.610
3	2.874	1.617-5.107	<0.001	2.954	0.837-10.432	0.092
**Metastasis at time of diagnosis**						
No	1.000			1.000		
Yes	2.842	1.855-4.354	<0.001	2.101	0.901-4.898	0.086
**Resection margins**						
Wide	1.000			1.000		
Non-wide	2.523	1.706-3.730	<0.001	2.245	1.077-4-680	0.031
**Ki67**						
Low	1.000		0.432*	1.000		
Medium	1.059	0.528-2.163	0.876	-	-	-
High	1.365	0.710-2.625	0.351	2.553	1.167-5.584	0.019
**M-CSF**						
Low	1.000			NIA		
High	0.815	0.463-1.435	0.478			
**TGF-beta**						
Low	1.000			NIA		
High	0.682	0.247-1.881	0.460			
**M-CSF and TGF-beta**						
Low	1.000			NIA		
High	1.532	1.062-2.208	0.022			

* Overall significance as a prognostic factor. NIA = Not included in analysis**.**

In patients with tissue from peritumoral capsule, independent negative prognostic variables were non-wide resection margins (P = 0.031) and high expression of Ki67 (P = 0.019, Table 6)

## Discussion

In this study we evaluated whether there is an association between the expression of CD57, CD68, M-CSF, CSF-1R, Ki67 and TGF-beta in tumors or peritumoral capsule and survival in 249 non-GIST STS patients. Increased co-expression of M-CSF and TGF-beta in the tumor and increased expression of Ki67 in the peritumoral capsule were independent negative prognostic factors for DSS in patients with STS. High expression of M-CSF in tumor was correlated with high malignancy grade, increased Ki67 and short DSS. To our knowledge, this is the first report on co-expression of M-CSF and TGF-beta in STS and the first evidence of its possible clinical relevance in STS patients.

STS have varying biological characteristics regardless of histological entities. Its prognosis is poor, but also difficult to predict. This aggressive behavior reflects, at least in part, the capacity of the tumor to evade host immune surveillance. Evasion strategies can protect cancer cells from immune responses by a variety of mechanisms including self-tolerance, sequestration of tissue from surveillance, antigen shedding, lymphocyte killing, secretion of immunosuppressive cytokines, lack of MHC II expression, lack of co-stimulatory molecules and local secretion of prostaglandins.

CD57 positive cells have been implicated in the resistance against malignant and virally-infected cells. Presence of these cells was observed to be an independent prognostic marker for a better DSS in squamous cell carcinoma [31] and adenocarcinoma [32] of the lung, as well as in other cancers such as colonic and gastric carcinomas [14,15]. In NSCLS, high density of stromal CD57 positive cells was an independent, positive prognostic factor for DSS, whereas high density of CD57 positive cells within neoplastic cell areas was not [33]. In our material there was no such correlation in tumor or peritumoral capsule. The location of infiltrating lymphocytes may be important. There are major differences between 1) inflammatory cells within cancer cell nests in carcinomas (epithelial CD57 positive cells); 2) inflammatory cells present in the stroma of epithelial tumors (stromal CD57 positive cells), 3) inflammatory cells present along the invasive margins (peritumoral CD57 positive cells); and, 4) inflammatory cells in the peritumoral capsule of stromal tumors such as STS.

In addition to NK-cells, expression of CD57 is also found on T-lineage lymphocytes, where it is currently considered a marker-replicative senescence (“clonal exhaustion”), i.e., a high susceptibility to activation-induced cell death and the inability to undergo new cell-division cycles despite preserved ability to secrete cytokines upon encounter with their cognate antigen [34]. Even on NK cells it does not constitute a one-marker-labels-all solution: CD57 defines a functionally distinct population of mature NK cells in the human CD56dim CD16+ NK-cell subset [35].

Studies have demonstrated a close association between M-CSF and tumor progression in lung cancer cell lines [36]. In a NSCLC cohort studied by Kaminska et al. [37], high pretreatment serum levels of M-CSF were an independent predictor of poor survival in these patients. However, Al-Shibli et al. [33] did not find any correlation between expression of M-CSF in NSCLC and DSS. CSF-1 protected osteoclasts from suppressive effects of transforming growth factor beta (TGF-beta) in a mouse mammary tumor cell line [38]. Kirma et al. studied M-CSF and TGF-beta in cervical cancer and found that CSF-1R (c-fms proto-oncogene product) activation may play a role in cervical carcinogenesis [39]. Richardsen et al. [27] showed that high M-CSF expression was correlated with a high malignancy grade in STS. In our study, high M-CSF expression in tumor correlated with a high malignancy grade, increased Ki67 and DSS in univariate analyses. But the expression of M-CSF in peritumoral capsule showed no correlation with DSS.

TGF-beta is a multifunctional cytokine known to induce G1 arrest in order to end proliferation, induce differentiation, or promote apoptosis in normal cells, thus being a natural tumor-suppressive agent. Though in tumorigenesis this mediator initiates EMT through activation of Smad and non-Smad signalling pathways[40]. Such pro-neoplastic action becomes possible through either blockade of the TGF-beta pathway with receptor-inactivating mutations, or selective inactivation of the tumor-inhibiting arm of this pathway[41]. High TGF-beta expression was an independent negative prognostic factor for disease specific survival in STS[42]. In the multivariate analysis, co-expression of M-CSF and TGF-beta were an even stronger negative prognostic factor in this study. We found a co-variation of expression of M-CSF and TGF-beta in tumor. TFG-beta might regulate the expression of M-CSF. Grayfer et al. reported on the regulation of pro-inflammatory functions of goldfish macrophages and induction of gene expression by recombinant goldfish CSF-1 (rgCSF-1). At 72 h post treatment rgCSF-1 increased the expression of TGF-beta [43]. The combined expression of immunostimulatory granulocyte macrophage colony stimulating factor (GM-CSF) and antitumor suppressor TGF-β2 antisense (AS) transgenes can break tolerance and stimulate immune responses to cancer-associated antigens which make it possible to design bifunctional therapeutic anti-cancer vaccines[44].

Increased expression of Ki67 and M-CSF in tumor are negative prognostic indicators for patients with STS, but this is not independent of malignancy grade. In the univariate analysis presented TGF-beta seems to be the dominating factor, while low or high M-CSF expression in combination with low TGF-beta expression does not seem to influence prognosis significantly. Both expression of TGF-beta and M-CSF have co-variation with malignancy grade and expression of Ki67. In the multivariate analysis the co-expression of M-CSF and TGF-beta was a stronger prognosticator for DSS than each of the markers alone. Expression of Ki67 in tumor was not an independent prognosticator. As mitotic activity is one of the criteria determining the malignancy grade, expression of Ki67 is closely correlated to mitotic activity, hence also malignancy grade [45]. Archad et al. found that malignancy grade is a more important prognostic factor in glial neoplasms than Ki67 [19]. So Ki67 may not provide additional information if the tumor malignancy grade is classified correctly. The tumor stroma is important for cancer progression [46]. There is no evaluation of tumor stroma in the grading systems of STS. But Ki67 expression in the peritumoral capsule may have prognostic impact in addition to malignancy grading of the tumor. Further research is needed to determine whether an increased expression of Ki67 may be the result of on increased migration of fast-proliferating cells in the peritumoral capsule or an enhanced proliferation effect of tumor-released cytokines on the stromal cells.

## Conclusion

In summary, increased co-expression of M-CSF and TGF-beta in tumor and increased Ki67 expression in the peritumoral capsule of STS patients were independent negative prognostic factors for DSS. This data may provide additional information to guide therapy after surgical resection.

## Competing interests

The authors declare that they have no competing interests.

## Authors’ contributions

SWS, TK, AV, TD, RMB and LTB participated in the design of the study. TK and AV collected clinical information. SWS and AV reviewed all the histological diagnosis, histological grading, selected and marked the slides for TMA construction. SWS, TK and AV performed the experiments. SWS, TK, AV, TD, RMB and LTB performed the statistical analysis. SWS, TK, AV, TD, ES, KAS and LTB contributed reagents/materials/analysis tools. SWS, TD, ES, KAS, RMB and LTB drafted the manuscript. All authors read and approved the final manuscript.

## Pre-publication history

The pre-publication history for this paper can be accessed here:

http://www.biomedcentral.com/1472-6890/12/7/prepub
